# A Gaze Independent Brain-Computer Interface Based on Visual Stimulation through Closed Eyelids

**DOI:** 10.1038/srep15890

**Published:** 2015-10-29

**Authors:** Han-Jeong Hwang, Valeria Y. Ferreria, Daniel Ulrich, Tayfun Kilic, Xenofon Chatziliadis, Benjamin Blankertz, Matthias Treder

**Affiliations:** 1 Machine Learning Group, Berlin Institute of Technology (TU Berlin), Marchstrasse 23, 10587 Berlin, Germany; 2 Neurotechnology Group, Berlin Institute of Technology (TU Berlin), Marchstrasse 23, 10587 Berlin, Germany; 3 Behavioural & Clinical Neurosciences Institute, Department of Psychiatry, University of Cambridge, UK

## Abstract

A classical brain-computer interface (BCI) based on visual event-related potentials (ERPs) is of limited application value for paralyzed patients with severe oculomotor impairments. In this study, we introduce a novel gaze independent BCI paradigm that can be potentially used for such end-users because visual stimuli are administered on closed eyelids. The paradigm involved verbally presented questions with 3 possible answers. Online BCI experiments were conducted with twelve healthy subjects, where they selected one option by attending to one of three different visual stimuli. It was confirmed that typical cognitive ERPs can be evidently modulated by the attention of a target stimulus in eyes-closed and gaze independent condition, and further classified with high accuracy during online operation (74.58% ± 17.85 s.d.; chance level 33.33%), demonstrating the effectiveness of the proposed novel visual ERP paradigm. Also, stimulus-specific eye movements observed during stimulation were verified as reflex responses to light stimuli, and they did not contribute to classification. To the best of our knowledge, this study is the first to show the possibility of using a gaze independent visual ERP paradigm in an eyes-closed condition, thereby providing another communication option for severely locked-in patients suffering from complex ocular dysfunctions.

A brain-computer interface (BCI) is a communication method that translates brain signals into commands for controlling external devices. It can thereby provide an alternative communication channel for severely paralyzed patients, such as amyotrophic lateral sclerosis (ALS) patients. To develop BCI systems based on event-related potentials (ERPs), various sensory modalities have been exploited^1^, i.e., vision, hearing, and somatic sensation, in which the user is asked to attend on one of the external stimuli, and brain signals evoked by different stimuli are discriminated and used as an input source for controlling BCI systems. In particular, vision-based BCI paradigms have been intensively studied^1^ because they generally provide a more intuitive way of mapping stimuli to commands accompanying higher communication performance. Moreover, they typically offer a better classification performance in multi-class BCI systems, compared to auditory and haptic BCI paradigms^2^,^3^,^4^,^5^.

Two widely used visual BCI paradigms are steady-state visual evoked potential (SSVEP) and visual ERPs^1^. SSVEP is a periodic brain response to a visual stimulus flickering at a certain frequency. A visual ERP is a brain response that is phase-locked to the presentation of visual stimuli, e.g., oddball paradigm. Both paradigms have been widely used in developing a variety of BCI applications, e.g., mental speller^6^,^7^, wheelchair navigation^8^, prosthesis control^9^, and mouse cursor control^10^, and shown the promising possibility of using BCI systems in daily life situations. For example, mental spellers based on each paradigm showed a typing speed of up to 10 letters/min^7^,^11^, and recent studies further improved the typing performance of BCI spellers by hybridizing the SSVEP and visual ERP paradigms^12^,^13^,^14^.

However, patients with neurodegenerative diseases who are the main targets of BCI technology gradually lose their motor functions, including decline of visual functions in progressed states of the disease^15^,^16^,^17^,^18^,^19^,^20^,^21^,^22^. Several types of dysfunctions in oculomotor control have been reported in the patients, e.g., gaze palsy^19^,^22^, slow saccade^17^,^19^,^22^, nystagmus^15^,^16^,^20^, and eyelid dropping (ptosis)^18^,^23^. Since conventional visual BCI systems require moderate eye movements to gaze at a target during stimulation, patients suffering from these symptoms cannot take full advantage of the conventional BCI systems. For those with impaired oculomotor functions, a gaze independent BCI paradigm was introduced, where the subject covertly focuses on a target stimulus while gazing at the center of a screen without eye movements^6^,^24^,^25^,^26^,^27^,^28^,^29^,^30^,^31^. Also, a recent study first showed the feasibility of an eyes-closed visual BCI paradigm based on SSVEP under overt attention condition^32^.

However, existing gaze independent BCIs have limited application value if oculomotor impairments are severe. For instance, a classical gaze independent BCI system can be used for patients with gaze palsy, but not with involuntary eyelid dropping or slow blink because it is required for successful operation that the subject should stably keep the eyes open to covertly recognize visual stimuli using peripheral vision. Also, patients with gaze palsy and slow saccade cannot successfully utilize the recently proposed eyes-closed visual BCI paradigm requiring direct gaze at a target, and thus the proposed paradigm can be especially applied to patients suffering from ocular ptosis or low blink rates, but having mild gaze function at least^32^.

In the present study, we propose a novel gaze independent visual BCI paradigm based on ERPs that are modulated by visual stimulation through closed eyelids, so that it potentially applies to locked-in state (LIS) patients with complex oculomotor impairments and completely locked-in (CLI) patients. To verify the feasibility of the proposed BCI paradigm, a visual stimulation system was implemented using a pair of glasses and four LEDs with which online BCI experiments were conducted with twelve healthy subjects. In the online experiment, visual stimuli were presented to the subjects with eyes closed while wearing the glasses-based stimulation system, and they were asked to covertly attend on one of the stimuli without directly gazing at a target in order to answer given questions. Classification outputs were given to the subjects for each trial in real-time. Further analyses on classification accuracy and ERPs were performed offline.

## Method

### Subjects

Twelve healthy subjects participated in this study (8 males and 4 females, aged 30.41 ± 3.39 s.d. years). Three had previous experiences with BCI experiments, and the others were naïve with respect to BCIs. None had a history of neurological, psychiatric or other severe disorders that might affect experimental outcomes. All subjects had normal or corrected-to-normal vision. The fundamental goal of this study and detailed experimental procedures were explained to each subject, and then they signed consent forms before the experiments. This study was approved by the Ethics Committee of the Institute of Psychology and Ergonomics, Technical University of Berlin (approval number SH_01_20150330), and all experiments were conducted in accordance with the declaration of Helsinki.

### EEG Data Recording

During the experiments, EEG signals were sampled at 1000 Hz using a multi-channel EEG acquisition system (BrainCap, Brain Products, Munich, Germany) with 63 scalp electrodes placed according to the international 10–10 system. The electrode locations were Fp1–2, AF3–4, 7–8, Fz, F1–10, FT7–8, FCz, FC1–6, T7–8, Cz, C1–6, TP7–8, CPz, CP1–6, Pz, P1–10, POz, PO3–4, 7–8 and Oz, O1–2. Two EOG channels were created by bipolarly referencing two pairs of electrodes (horizontal EOG channel: F9–F10; vertical EOG channel: (Fp1+Fp2)/2). The EEG signals were referenced to the left mastoid with a forehead ground. The hardware bandpass filter with cutoff frequencies of 0.016 and 250 Hz was applied before the sampling. The impedance of all electrodes was kept below 20 kΩ.

### Visual Stimulation

**Figure 1:**
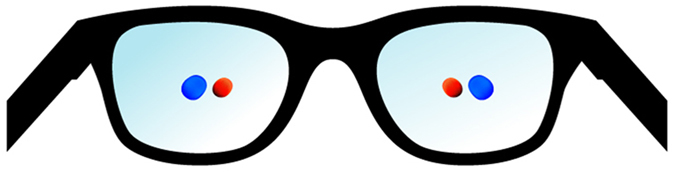
Schematic illustration of the developed visual stimulation system. Two LEDs colored with blue and red were attached with a distance of 1 cm on the center of each glass. The red LEDs placed inside of each glass were paired and each blue LED was separately employed, such that a 3-class BCI system was implemented. The duration of a single flash and inter-stimulus interval (ISI) were set to 100 ms and 1200 ms, respectively.

To present visual stimuli in eyes-closed condition, we constructed a visual stimulation system which consists of a pair of glasses, four LEDs, and an LED controller. An Arduino Leonardo board containing an ATmega32u4 microprocessor was used as the LED controller. As sketched in Fig. 1, two LEDs emitting blue and red light were attached on each side of the glasses for which we drilled two holes on each glass, inserted the LEDs, and fixated them using glue. In order to realize a 3-class BCI system, the two red LEDs placed in the middle were paired and synchronously employed, and the blue LEDs on the left and right side of each glass were independently flashed. The duration of a single flash was 100 ms, and inter-stimulus-interval (ISI) was set to 1200 ms. The relatively long ISI compared to typical ERP studies was empirically determined to help the subjects perceive an upcoming stimulus from the current one in terms of color because the visual stimulus with a short distance from the eyes (<5 cm) yielded longer afterimage durations than in ordinary stimulation conditions. The luminance intensity of the LEDs was empirically selected through preliminary experiments, which were 95 and 80% of the original luminous intensity for the red (500 mcd/20 mA) and blue (1000 mcd/20 mA) LEDs, respectively. With this stimulation setting, none of the subjects reported feeling uncomfortable or having difficulties focusing on a target stimulus.

### Questions

In the experiments, to demonstrate the possibility of using our proposed BCI paradigm in real clinical situations, questions having three possible choices were automatically read out to the subjects using a realistic speech synthesizer. Subjects were asked to answer the questions by concentrating on one of the three different types of visual stimuli, e.g., “Which of these drinks is alcoholic?” a) coffee, b) lemonade, c) beer. Each of ‘a’, ‘b’, and ‘c’ options corresponded to the left blue LED, the right blue LED, and the pair of middle red LEDs. Different questions were used for each trial, and the order of the questions was randomized for each subject.

### Experimental Procedures

Subjects were sitting on a comfortable chair, and wearing the LED-attached glasses after EEG preparation. They were asked to avoid any body movements during the experiments and to keep their eyes shut during visual stimulation. This was continuously monitored, and subjects neither made considerable body movements nor did they open their eyes during the experiment. The visual stimuli were first presented sequentially without EEG recording to check whether the subjects could recognize the position (left, middle or right) and color (blue or red) of each LED stimulus. The visual stimuli were presented to the subjects until they fitted the position of the LED glasses as comfortably as they could perceive each stimulus, which was generally done within several iterations. Each experiment consisted of one calibration and three feedback sessions. In the calibration session, data was collected to construct a subject-specific classifier. Fifteen questions (trials) and their true answers were presented, and subjects had to covertly attend to the LEDs corresponding to the designated true answers. Subsequently, 3 feedback sessions with 20 questions each were conducted (a total of 60 questions). Subjects were prompted to choose answers to each question by themselves, focus on self-selected LEDs during visual stimulation, and input their answers using a keyboard after each trial. The numbers 1–3 were used for answering ‘a’, ‘b’, ‘c’ options, respectively, for which the subjects fixed their index, middle, and ring fingers on the numbers 1–3 of the keyboard during the whole feedback sessions. A classifier output (‘a’, ‘b’, or ‘c’) was acoustically given as feedback right after the subject input his/her own answer, and online classification accuracy was calculated by comparing the classifier output with the subject’s answer. For visual stimulation, the three groups of LEDs were randomly illuminated eight times for each trial in both calibration and feedback sessions (3 groups x 8 sequences = 24 flashes). Thus, the time required for one selection was 31.2 s (1,300 ms x 24 flashes = 31.2 s). A break of about 5 minutes was given between the sessions. Subjects reported that the experimental task was not too hard.

### EEG Data Analysis

All online and offline data analyses were performed after downsampling to 100 Hz. No software filter was applied for online data analysis. A linear discriminant analysis (LDA) classifier with shrinkage of the covariance matrix was used for online classification during the feedback session^33^. To train a shrinkage LDA classifier, the calibration data were first epoched from – 200 ms to 1000 ms based on the stimulus onset time, and then baseline correction was performed using the data 200 ms prior the stimulus. Epochs and channels containing physiological artifacts (e.g., eye and muscle movements) were removed based on a variance criterion. About 4% of epochs and less than 1 channel were rejected on average. Then, the most discriminative five temporal intervals were selected using a heuristic search based on signed square values of point-biserial correlation coefficients (*sgn r*^2^), and the channel-wise mean amplitudes in the selected time intervals were calculated as features. The shrinkage LDA was trained using the features, and applied to the data measured during the feedback sessions for online classification (see Blankertz *et al*.’s study^33^ for the data processing pipeline in detail).

Three different offline analyses were performed to investigate ERPs elicited by the visual stimuli presented in eyes-closed condition and to demonstrate the possibility of using our proposed eyes-closed BCI paradigm in covert attention condition, respectively. Before all offline data processing, the recorded EEG data were first lowpass filtered below 49 Hz using a Chebyschev filter with passbands and stopbands of 42 and 49 Hz to remove powerline interference. The first offline analysis was to investigate ERPs for which the data sets measured from the three feedback sessions were used (60 trials) and the first three steps of the method used in training the shrinkage LDA classifier was identically applied to the data sets (data epoching, baseline correction, and artifact rejection). Since the ERP analysis results showed stimulus-specific eye movements (see the Supplementary Figures S1 and S2 in advance), we performed a second offline analysis in order to investigate the contribution of eye movements to classification. The horizontal and vertical EOG channels were used to examine stimulus-specific eye movements, and offline classification accuracy was estimated with three different channel sets (all channels, six frontal channels (Fp1–2, AF3–4,7–8), and the other channels) to check the spatial and temporal distribution of discriminative information, especially for the frontal electrode set. It was assumed that the six frontal electrodes contain the information most pertinent to eye movements based on the ERP topographic maps shown in the Supplementary Figures S1 and S2. For the latter analysis, a standard binary classification (target vs. non-target; chance level 50%)^33^ was separately performed for each electrode set by taking whole temporal features (yielding a spatial distribution of discriminative information), and for each time interval created by a 80 ms sliding window with 50% overlap (yielding a temporal distribution of discriminative information) in which the three sets of electrodes were also separately employed. In the third offline analysis, we calculated classification performance after clearly removing all identifiable physiological artifacts, especially eye movements, to further check the impact of stimulus-specific eye movements on classification. To obtain cleaned EEG signals, the original EEG data were decomposed into neural and artifactual source components by using independent component analysis (ICA), and artifactual components were projected out. An artifactual independent component classification method, called MARA (Multiple Artifact Rejection Algorithm)^34^,^35^ was used to automatically select artifactual components. Using the artifact-free EEG data, online classification was simulated in offline fashion with the identical method employed for the online classification. In this study, a Matlab toolbox, EEGLAB, and its plug-in, MARA, were used to perform ICA and MARA, respectively (http://www.user.tu-berlin.de/irene.winkler/artifacts/).

For statistical analysis, two non-parametric methods, Friedman and Wilcoxon signed-rank test, were performed because testing data sets did not follow a normal distribution as confirmed by the Kolmogorov-Smirnov test. The Friedman and Wilcoxon signed-rank tests correspond to the parametric statistical tests, one-way repeated-measures ANOVA and paired t-test, respectively. The significance level for the Friedman test was set to 0.05, and a Bonferroni-adjusted significance level was used for the Wilcoxon post-hoc analysis, i.e., *p* = 0.05/the number of post-hoc tests.

## Results

### Online Classification and ERPs

**Figure 2:**
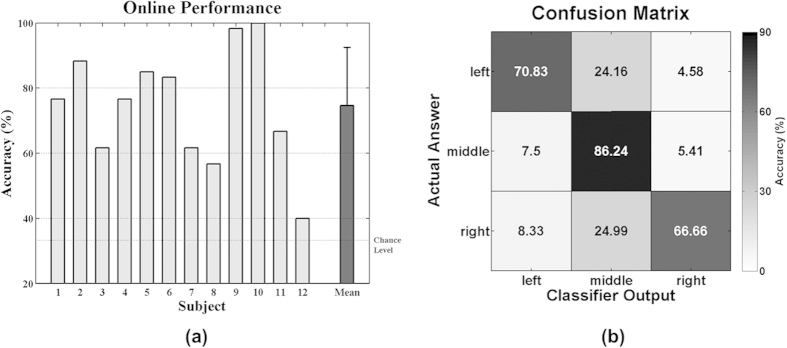
Online classification results. (**a**) Classification accuracies of each subject, and their mean and standard deviation (chance level 33.33%). (**b**) Confusion matrix of online classification results. The mean classification accuracies of the left, middle, and right class are 70.83, 86.24, and 66.66%, respectively, and most false negatives of the left and right class are observed in the middle class (24.16% for the left class and 24.99% for the right class, respectively).

Figure 2(a) shows the online classification accuracies of each subject and their mean. All subjects achieved performance substantially higher than the chance accuracy of 33.33%, with a mean accuracy of 74.58% across subjects. Figure 2(b) shows the confusion matrix of the online classification results. The correct mean recognition rates of left, middle, and right targets were 70.83%, 86.24%, and 66.66%, respectively, and the performance of middle targets is significantly higher than that of both left and right ones (Friedman χ^2^(2) = 8.54, *p *= 0.014; the Bonferroni post hoc analysis: middle > left = right, corrected *p* < 0.01). It is also observed in Fig. 2(b) that most false negatives for the left and right class occur in the middle class (24.16% and 24.99% false negatives for left and right targets, respectively).

**Figure 3:**
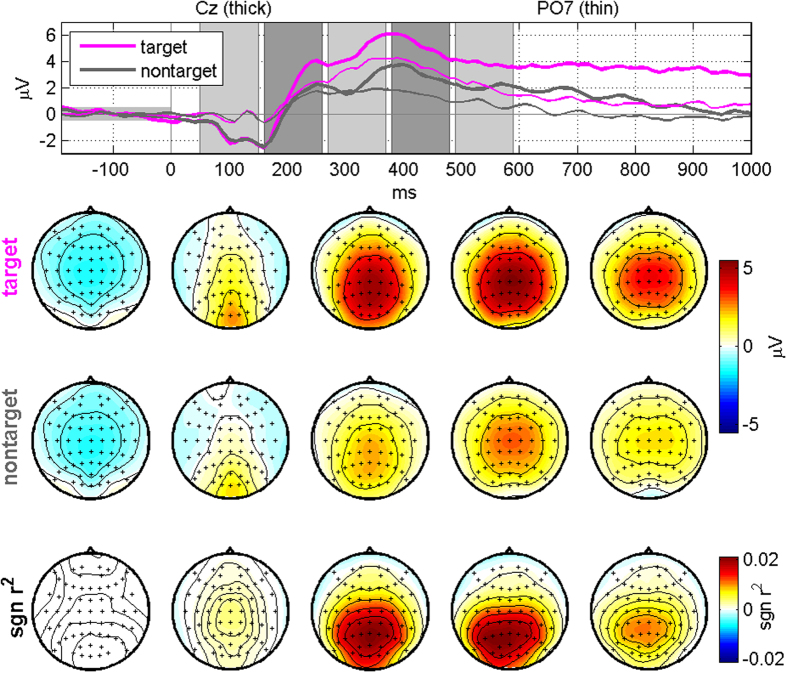
Grand-average ERPs for target and non-target stimuli and their differences in terms of the *sgn r*^*2*^ value along time after removing artifact components using ICA. No significant eye movements are observed in the ERP maps, while P3 components are clearly seen. The topographic maps in each column correspond to the five time periods shaded in the top panel.

**Figure 4:**
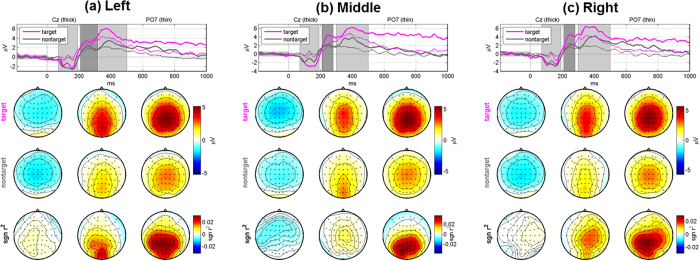
Class-specific grand-average ERPs for target and non-target stimuli and their differences in terms of the *sgn r*^*2*^ value after removing artifact components using ICA when a target is the (**a**) left, (**b**) middle, and (**c**) right stimulus, respectively. P3 components more shifted to the direction of a visual stimulus are observed in the *sgn r*^*2*^ maps of the second time periods for left and right targets. In each figure, the topographic maps in three columns correspond to the three time periods shaded in the top panels, and the time intervals were empirically selected to better show the target-specific ERP patterns.

Figure 3 depicts grand-average ERPs of the cleaned EEG signals obtained after applying ICA for target and non-target stimuli and their differences in terms of the *sgn r*^*2*^ value, and Fig. 4 separately shows grand-average ERP topographical maps for each target. In these figures, typical P3 components are seen in both target and non-target conditions, but they are considerably larger for targets than for non-targets (see the Supplementary Figures S1 and S2 for the grand-average ERPs of the original EEG data before artifact rejection).

### Eye Movements

**Figure 5:**
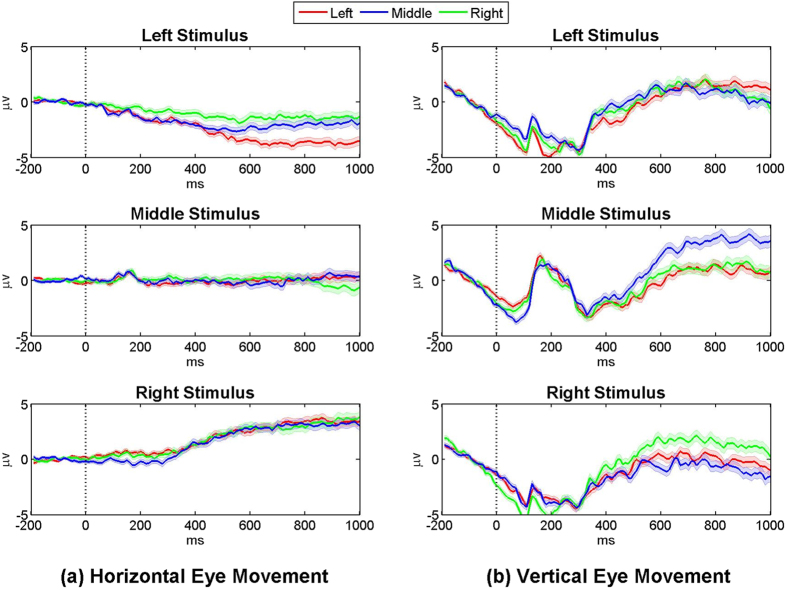
(**a**) Horizontal and (**b**) vertical eye movements occurred during stimulation. The red, blue, and green lines indicate eye movements produced when a target is the left, middle, and right stimulus, respectively, and the shaded areas of each line represent the standard errors of respective EOG signals. Irrespective of whether visual stimuli are targets or non-targets, eye movements moved to the opposite direction of a visual stimulus are confirmed in horizontal EOGs when left and right stimuli are presented, while dominant vertical EOGs are observed for middle stimuli.

Along with the P3 components, eye movements are also found as evidenced by activity in frontal electrode sites (see Supplementary Figures S1 and S2). Figure 5 shows the characteristics of the eye movements induced by each directional stimulus, where horizontal and vertical EOGs are separately presented. The red, blue and green lines represent EOGs measured when a target is left, middle, and right LED, respectively, for each stimulus. It is confirmed in Fig. 5(a) that the subjects shifted their eyes to the opposite side of a visual stimulus when left and right stimuli are presented, irrespective of whether they are targets or non-targets (see the first and third rows in Fig. 5(a)). Note that because horizontal EOG was calculated by subtracting F10 from F9, a negative EOG value corresponds to a shift of the eyes to the right and vice versa. Little horizontal movements are observed for the middle stimulus but strong vertical eye movements are shown, while the left and right stimuli induce little vertical movements (see around 200 ms in Fig. 5(b)). The stimulus-specific eye movements are in line with those observed in the grand-average ERP maps of the original EEG data (Supplementary Figure S2).

### Offline Classification

**Figure 6:**
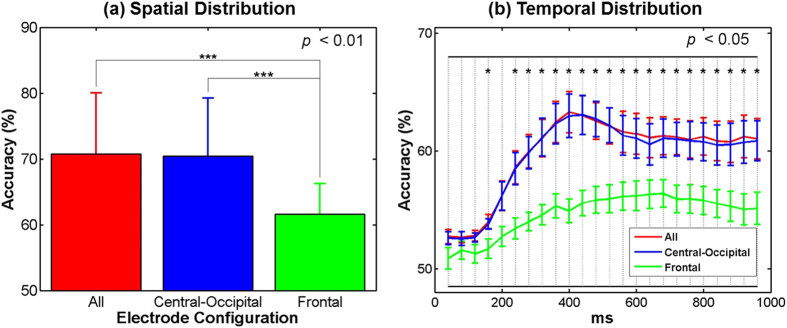
(**a**) Spatial and (**b**) temporal distribution of discriminative information of three electrode sets. The electrode sets, “All”, “Frontal”, and “Central-Occipital”, were constructed using all electrodes, six frontal electrodes (FP1–2, AF3–4, 7–8), and the others excluding the frontal electrode set, respectively. In general, the performance of the ‘Frontal’ electrode set is significantly lower than that of the other electrode sets. The vertical bars represent the standard deviations of the classification accuracies for each condition.

The spatial and temporal distribution of discriminative information of the three electrode sets is presented in Fig. 6. Significantly lower performance is consistently observed in the frontal electrode set (“Frontal”) in which eye movements are most strongly reflected, compared to the other two electrode sets (‘All’ and “Central-Occipital”). In particular, no considerable difference in classification accuracy is observed between the two electrode sets constructed using all electrodes (“All”) and the central-occipital electrodes (“Central-Occipital”). The performance differences between the three electrode sets are statistically confirmed for both spatial (Fig. 6(a), Friedman χ^2^(2) < 32.08, *p *= 0.0007; the Bonferroni post hoc analysis: “All” = “Central-Occipital” > “Frontal”, corrected *p *< 0.01) and temporal distribution (Fig. 6(b), Friedman χ^2^(2) 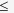
8.17, *p *
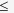
0.0169; the Bonferroni post hoc analysis: “All” = “Central-Occipital” > “Frontal”, corrected *p* < 0.05 for all time intervals except the first, second, third and fifth ones).

**Figure 7:**
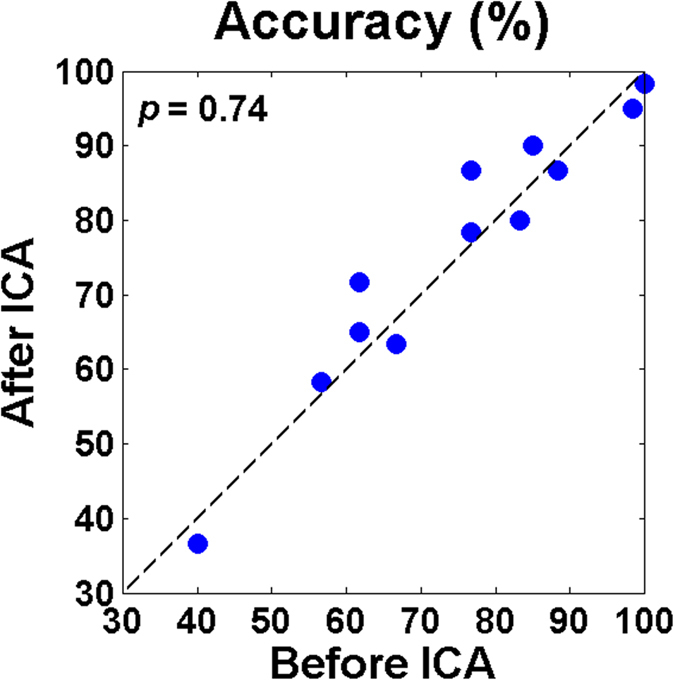
Comparison of simulated classification accuracies attained before and after applying ICA. The difference between the accuracies is not statistically significant (before ICA: 74.58% vs. after ICA: 75.83%, p = 0.74 with Wilcoxon signed rank test).

Figure 7 shows the comparison of classification accuracies obtained before and after artifact rejection using ICA for each subject. The degree of performance change varies from one subject to another, but it is not significantly different in average (before ICA: 74.58% vs. after ICA: 75.83%, *p *= 0.74 with Wilcoxon signed rank test). The confusion matrix of the simulated classification results (after ICA) showed a similar trend to that of original ones (before ICA) in terms of classification accuracy of each class and false negatives of left and right targets (not shown here).

## Discussion

Visual BCI paradigms have been intensively studied to realize practical BCI systems for paralyzed patients, but the performance of conventional visual BCI systems decreases significantly when users are not allowed to directly gaze at a target stimulus^24^,^36^,^37^. Even though variations of the classical paradigm have been introduced to overcome clinically relevant problems (e.g., gaze independent or eyes-closed paradigms), they cannot be also applied to severely locked patients with multiple visual dysfunctions because they have been generally developed for a certain type of oculomotor impairment. In this study, we introduced a novel visual BCI paradigm that could be used in both gaze independent and eyes-closed conditions to encompass multiple oculomotor abnormalities, and demonstrated the feasibility of our novel BCI paradigm with a high mean online performance of 74.58%.

Since our proposed BCI paradigm was intended for LIS patients suffering from complex ophthalmoplegia and CLI patients, the characteristics of eye movements induced during visual stimulation should be carefully investigated. A series of analyses confirmed that the subjects tended to move their eyes to an opposite or avoidable direction from a visual stimulus (see Fig. 5). Thus, stimulus-specific eye movements can be explained by a reflex action to protect the eyes from a sudden light stimulus as pupillary light reflex. Most importantly, because the subjects consistently showed the reflex response for the same stimulus, irrespective of whether it is a target or non-target, eye movements did not contribute to classification performance (see Fig. 6). This suggests that the class-discriminative ERPs reflect genuine neural attention related processes. It was also reported in the literatures that ocular reflexes are generally weaken in patients with motor neuron diseases (MND)^38^,^39^, but saccade reflex similar to the reflexive eye movements observed in this study is relatively well preserved in those patients^40^,^41^,^42^,^43^. This is because the impairment of the frontal eye field that frequently happens in MND patients leads to eye movement abnormalities, but does not affect reflexive saccades in general^44^. This indicates that the potential target users of our proposed paradigm could also show reflexive eye movements similar to those shown in healthy subjects. Taken all results together, it can be expected that our paradigm might be useful for LIS patients suffering from multiple oculomotor impairments and CLI patients with cover attention, although this needs further evaluation in a clinical study.

In the ERP maps shown in Figs 3 and 4, visible P3 components were observed even when non-target stimuli were presented, but they were not as strong as those elicited by target stimuli. Similar P3 patterns were also observed in our previous BCI studies^6^,^45^ when a center speller was employed where both target and non-target stimuli were presented in the fovea similar to our proposed paradigm in this study. This phenomenon was explained by the fact that visual stimulation centered in foveal regions with the highest photoreceptor density lead to more involvement of neurons for visual processing, thereby resulting in the visible P3 components even for non-target stimuli^6^,^45^. It seems reasonable to assume that the P3 components elicited by non-target stimuli in this study can be generated by a similar mechanism.

**Figure 8:**
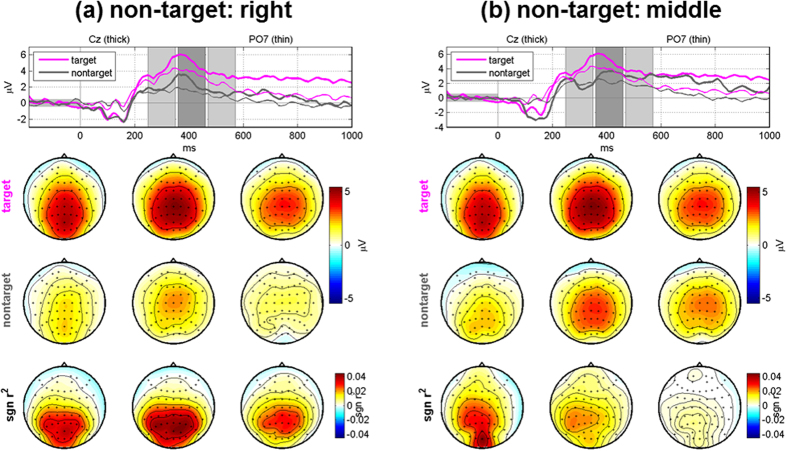
Grand-average ERPs for target and non-target stimuli and their differences in terms of the *sgn r*^*2*^ value after removing artifact components when a target is the left stimulus. The ERP maps are separately presented when a non-target stimulus is (**a**) the right LED and (**b**) the paired middle LEDs, respectively. The P3 components elicited by the middle non-target LEDs are stronger than those elicited by the right non-target LED, and thereby the differences of P3 components between targets and non-targets are reduced when middle non-target stimuli are presented, compared to right non-target ones. In each figure, the topographic maps in three columns correspond to the three time periods shaded in the top panels, and the time intervals were empirically selected between 250–570 ms to better show P3 components.

Most of misclassified left and right targets were assigned to the middle class, as shown in Fig. 2(b). This result can be interpreted by the mismatch of the number of LEDs used for each class along with the mentioned cortical magnification caused by the fovea-centered stimulation. In this study, a pair of LEDs was used for the middle stimulus and they were presented to both eyes simultaneously, while one LED was used for the left and right stimulus, respectively. As already discussed, relatively high P3 amplitudes were also seen for non-target stimuli due to the impact of the fovea-centered stimulation. Therefore, P3 amplitudes for the middle non-target stimulus employing 2 LEDs could be larger compared to the left and right non-target stimulus. This would result in a reduced difference of P3 amplitudes between targets and non-targets whenever the middle non-target stimulus is presented, thereby provoking misclassifications toward the middle class when targets are left and right LEDs. In fact, this speculation is indirectly backed by Fig. 8 showing grand-average ERP maps obtained when a target is the left LED, where the ERPs elicited by non-target right and middle LEDs are separately illustrated. As expected, P3 amplitudes are stronger for the middle LEDs than for the right LED, and the P3 amplitude differences between targets and non-targets are considerably reduced in the case that a non-target is the middle stimulus (see the second and third rows in Figs. 8(a) and (b)). A similar trend is also observed when a target is the right LED (not shown here). This suggests that the number of LEDs and light intensity require careful balancing across stimulus conditions. In particular, the LED intensity should be more carefully elaborated before applying our paradigm to real target patients because a light stimulus might negatively affect patients’ eyes in long-term use.

An eyes-closed visual BCI paradigm was first introduced based on SSVEP^32^, where EEG patterns induced by attending either left or right visual stimuli were classified. The eyes-closed SSVEP paradigm showed a good classification performance ranging from 81.3% to 96% in average (chance level: 50%), depending on stimulation time, and the corresponding information transfer rates (ITR) were 9.09–10.62 bits/min. Compared to the performance of our proposed paradigm, classification accuracy cannot be directly compared due to different chance levels (50% vs. 33.33%), but the average ITR of the eyes-closed SSVEP paradigm is much higher than that of our paradigm (1.23 bits/min). However, it should be noted that the eyes-closed SSVEP paradigm requires accurate horizontal eye movements to focus on either left or right stimulus, thereby limiting its application value for patients with severe oculomotor dysfunctions. On the other hand, our proposed eyes-closed ERP paradigm could be used in gaze independent condition, as demonstrated in our results (see Fig. 7). Therefore, if a paralyzed patient had moderate ocular functions, the eyes-closed SSVEP paradigm would be a better option than our paradigm in terms of the communication rate. If it is not the case, our proposed eyes-closed and gaze independent BCI paradigm could be a better choice for communication. Nevertheless, the relatively low ITR should be improved in the future studies by optimizing various experimental variables, such as flash duration, ISI, and the number of visual stimuli, for practical use. Another solution to increase the communication rate of the proposed ERP paradigm would be to take advantage of SSVEP features by incorporating the SSVEP paradigm to our paradigm, as in previous hybrid BCI systems combining the SSVEP and visual ERP Paradigm^12^,^13^,^14^.

Recently, significant advances have been made in the development of BCI systems based on non-visual sensory modalities such as auditory^46^,^47^,^48^,^49^,^50^,^51^ and tactile^52^,^53^,^54^, and they could be also utilized for patients with poor ocular functions because they are independent of oculomotor functions. Some ERP studies compared different sensory modalities and consistently showed the superiority of a visual paradigm over auditory or tactile ones with respect to P3 amplitudes^55^,^56^ and BCI performance^2^,^3^,^4^,^51^. However, it does not mean that a visual BCI paradigm is the best option for all end-users. Instead, a feasible and practical paradigm is highly dependent on the individual patient’s state of disease, which was demonstrated in several end-user studies conducted with different sensory BCI paradigms^57^,^58^,^59^,^60^. For example, a tactile modality showed better BCI performance than visual and auditory ones^57^, while a better performance was shown for a visual paradigm than an auditory one^58^. Thus, a user-centered BCI paradigm should be first investigated in practice before applying BCI technology to end-users. In this sense, our proposed BCI paradigm can provide another option for patients suffering from multiple ocular impairments, along with auditory and tactile paradigms. Furthermore, as some studies already demonstrated the positive impact of multisensory stimuli paradigms on BCI performance (e.g., visual + auditory)^2^,^3^,^61^, our novel visual BCI paradigm could be used simultaneously with other sensory paradigms to improve communication rate.

## Additional Information

**How to cite this article**: Hwang, H.-J. *et al*. A Gaze Independent Brain-Computer Interface Based on Visual Stimulation through Closed Eyelids. *Sci. Rep*. **5**, 15890; doi: 10.1038/srep15890 (2015).

## Supplementary Material

Supplementary Information
