# Supplementary material for: A Gaze Independent Brain-Computer Interface Based on Visual Stimulation through Closed Eyelids
Source: Sci Rep. 2015 Oct 29;5:15890. doi: 10.1038/srep15890 (PMC4625131; doi:10.1038/srep15890)

A Gaze Independent Brain-Computer Interface Based on Visual Stimulation through Closed Eyelids

Han-Jeong Hwang, Valeria YanKous Ferreria, Daniel Ulrich, Tayfun Kilic,

Xenofon Chatziliadis, Benjamin Blankertz, Matthias Treder

Supplementary Figures S1-S2

Supplementary Figure S1. Grand-average ERPs of the original EEG data before artifact rejection for target and non-target stimuli and their differences in terms of the *sgn* r^2 value along time. More dominant P3 components are observed for targets compared to non-targets. Eye movements are also found as evidenced by activity in frontal electrode sites. The topographic maps in each column correspond to the five time periods shaded in the top panel.

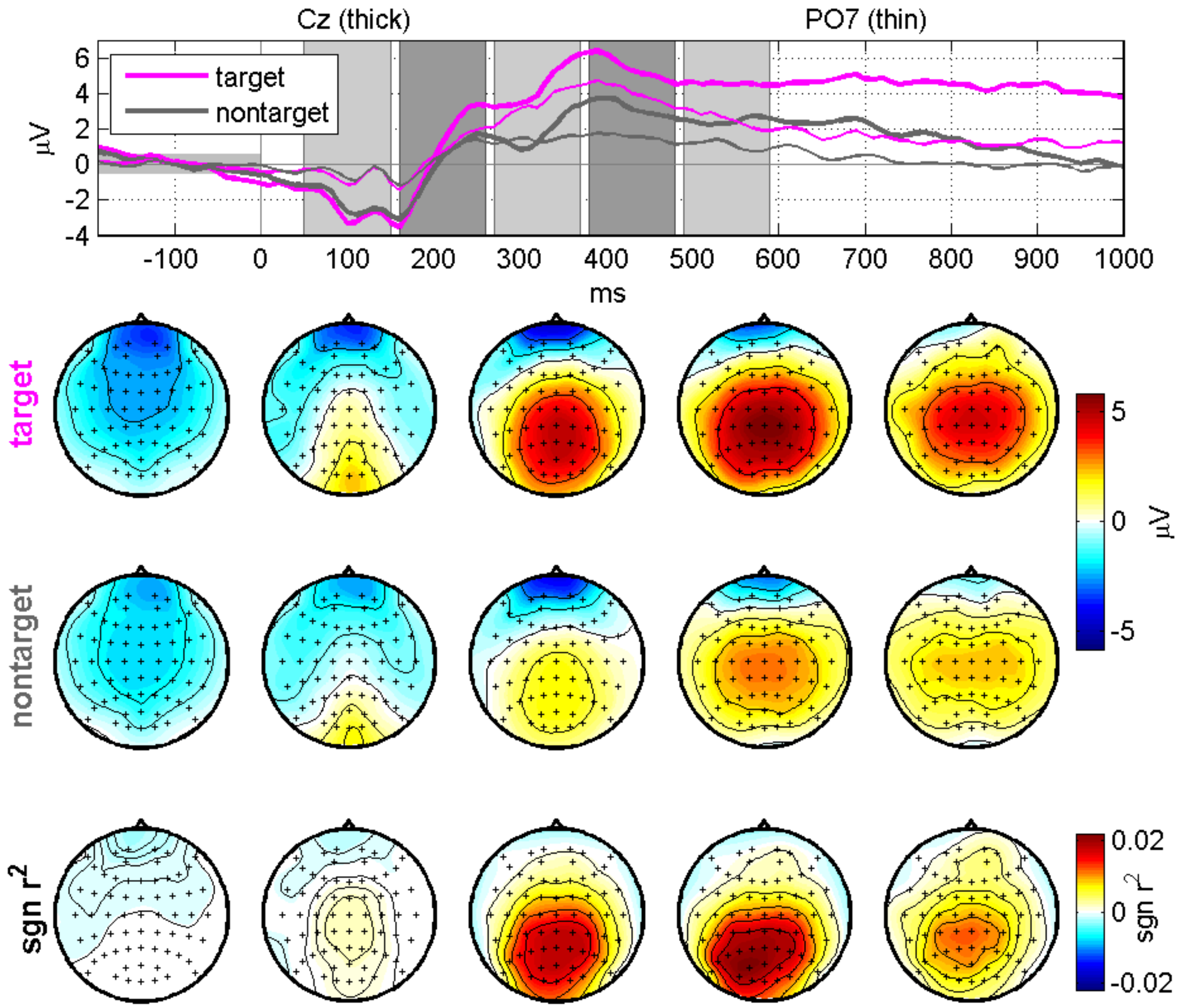

Supplementary Figure S2. Class-specific grand-average ERPs of the original EEG data before artifact rejection for target and non-target stimuli and their differences in terms of the *sgn* r^2 value for the (a) left, (b) middle, and (c) right target, respectively. Along with clear P3 components, the stimulus-specific eye movements are confirmed; that is, shifting the eyes opposite to the left and right stimulus (see the ERPs for targets in the third columns of (a) and (c)) and prominent vertical eye movements for the middle stimulus (see the ERPs for targets in the second column of (b)). In the mentioned columns of each figure, the same trend, EOGs more shifted to opposite sides for the left and right stimulus, is also observed in non-target ERP maps, even though they include the EOGs produced by the middle non-target stimulus (i.e., target: left vs. non-target: right and middle; target: right vs. non-target: left and middle). In the case that a target is middle, EOGs evenly spread to both left and right sides are shown in non-target ERP maps because EOGs responded to left and right stimuli are mixed. In each figure, the topographic maps in three columns correspond to the three time periods shaded in the top panels, and the time intervals were empirically selected to better show P3 components and stimulus-specific eye movements simultaneously.

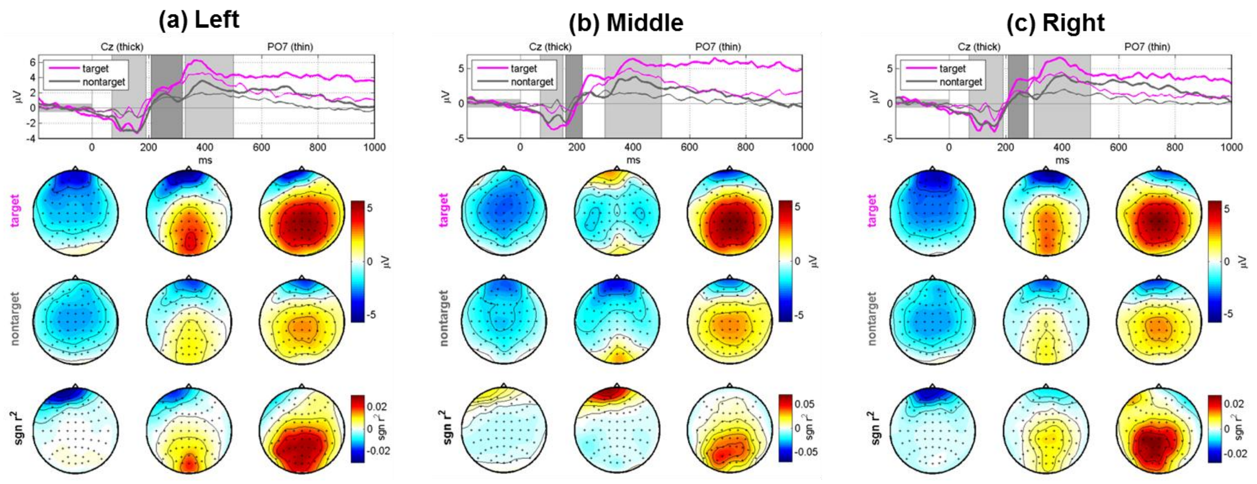

Supplement: Supplementary Information [file srep15890-s1.pdf]
